# The Heart as a Site of Metastasis of Benign Metastasizing Leiomyoma: Case Report and Review of the Literature

**DOI:** 10.1155/2018/7231326

**Published:** 2018-05-21

**Authors:** Mariam Meddeb, Robert D. Chow, Randolph Whipps, Reyaz Haque

**Affiliations:** Departments of Cardiology and Internal Medicine, University of Maryland Midtown Campus, University of Maryland Medical System, Baltimore, MD, USA

## Abstract

Uterine leiomyomas are the most common gynecological tumors in premenopausal women. While the lung is the most common extrauterine organ afflicted, benign metastasizing leiomyomas (BML) of the heart are rarities. We report an incidental finding of a cardiac mass in a 36-year-old woman who presented to the Emergency Department after a motor vehicle accident. CT scan of the chest revealed 2 well-circumscribed pulmonary nodules and a filling defect in the right ventricle. Echocardiogram showed a 4 cm mass attached to the right ventricular (RV) septum. The cardiac tumor was resected and showed benign histologic features. Immunohistochemical staining was positive for smooth muscle *α*-actin and desmin, as well as estrogen and progesterone receptors, consistent with the diagnosis of uterine leiomyoma.

## 1. Case

We present the case of a cardiac benign metastasizing leiomyoma incidentally found in a patient who presented to our hospital after a motor vehicle accident.

**Figure 1:**
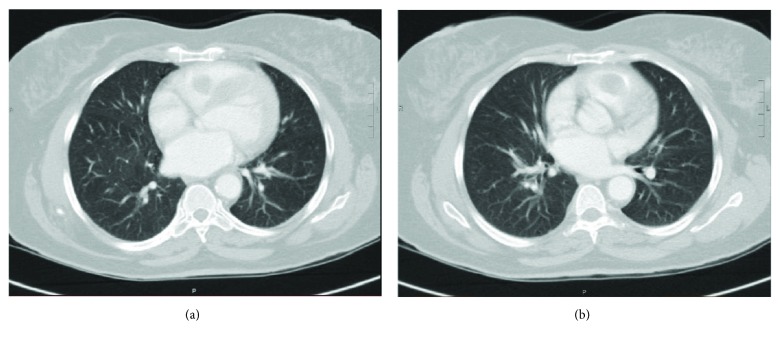
Chest CT scan with IV contrast showing a filling defect within the contrast-enhanced right ventricle which was initially thought to be a large thrombus extending to the right ventricular outflow tract.

**Figure 2:**
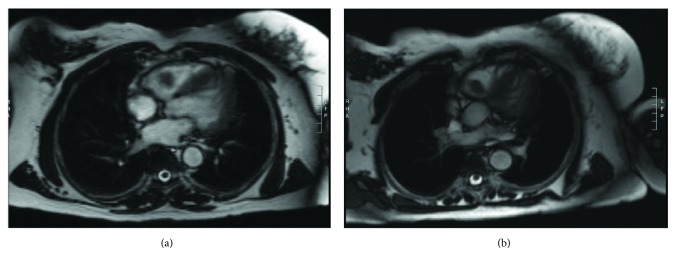
Cardiac magnetic resonance imaging showing an oblong soft tissue mass abutting the pulmonary valve.

A 36-year-old female patient presented to the emergency department of our hospital with right-sided blurry vision after being involved in a motor vehicle accident. The patient was a restrained back seat passenger and sustained a right eye trauma with no other injuries nor loss of consciousness. She was found to have traumatic uveitis. CT scan of the head and sinuses showed no evidence of fracture or acute pathology. A CXR was performed to rule out any rib fracture and found a 1.1 cm pulmonary nodule in the right midlung. A contrast CT scan confirmed the presence of 1.1 cm nodule within the anterior inferior aspect of the right upper lobe and found an additional 1 cm nodule within the posterior superior aspect of the right upper lobe. Both nodules were well circumscribed, compatible with benign nature. Unexpectedly, a large defect was noted within the contrast-enhanced right ventricle and was thought to be a large thrombus extending to the right ventricular outflow tract (Figure 1). The patient was started on therapeutic anticoagulation overnight. A transthoracic echocardiogram (TTE) and a subsequent transesophageal echocardiogram (TEE) disclosed the presence of a large mobile mass in the right ventricle arising from the interventricular septum. Turbulent flow was demonstrated in the right ventricular outflow tract (Supplemental Material video (available here)). Cardiac magnetic resonance imaging showed an oblong soft tissue mass that measured 4.9 cm in length abutting the pulmonary valve. The mass occupied the entire RV outflow tract (Figure 2).

The patient denied any symptoms including chest pain, dyspnea, syncope, or weight loss. Physical exam was significant for a systolic murmur. On further history taking, she reported that she underwent a hysterectomy 12 years earlier for “myomas” but denied any personal or family history of malignancy.

Abdominal/pelvic CT scan was performed and confirmed the absence of the uterus. No definite evidence of primary or metastatic disease was found with the exception of a 2.2 × 1.7 cm soft tissue nodule at the left aspect of the vaginal cuff, which was thought to be the left ovary. CA 125 level was within normal limits.

In the absence of evidence of primary malignancy, surgical resection of the cardiac tumor was pursued.

**Figure 3:**
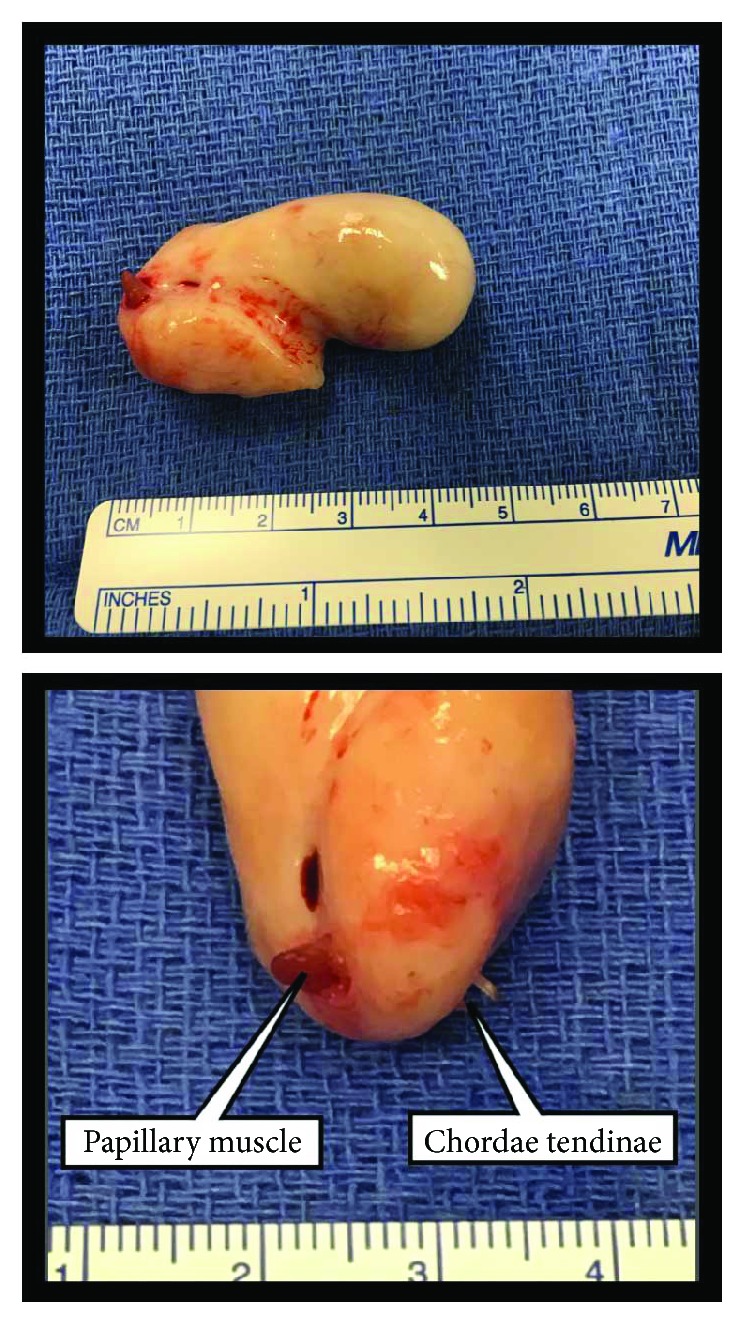
Surgical specimen consisting of a 12-gram pink-tan rubbery mass that is 4.7 × 2.6 × 2.1 cm. A scant amount of red-tan, shaggy adherent tissue is present on one edge representing the papillary muscle penetrated by the tumor. The sacrificed chordae tendinae traversing the lesion can also be seen on the other edge.

Intraoperatively, the mass was noted to be encircling the tip of a papillary muscle which was supporting the chordae to the anterior leaflet of the tricuspid valve. The mass was completely excised with sacrifice of the chordae tendinae traversing the lesion (Figure 3) and subsequent tricuspid valve repair (expanded polytetrafluoroethylene neochords and anteroposterior commissuroplasty).

Histologically, the tumor was composed of smooth muscle proliferation and a very low index of proliferation (Ki-67 < 10%), supporting a benign process. No atypia or necrosis was noted.

Immunohistochemically, tumor cells were strongly positive for smooth muscle *α*-actin, desmin, estrogen, and progesterone receptors, supporting a leiomyoma lineage.

The diagnosis of fibroma was excluded in light of the desmin stain positivity and elastin stain negativity. Immunostain for myogenin was negative, excluding the diagnosis of hamartoma of mature cardiac myocytes.

FDG PET-CT was performed postoperatively and revealed a faint FDG uptake within the 2 previously identified pulmonary nodules (SUV 1.4) and the soft tissue nodule on the left aspect of the vaginal cuff, later identified as parasitic leiomyoma by transvaginal ultrasound (US) and not the left ovary as initially thought.

No additional sites of metastasizing leiomyoma were identified.

The patient recovered from cardiac surgery without complications. She was subsequently started on aromatase inhibitor treatment (anastrozole). CT scan of the chest-abdomen-pelvis 3 months after surgery showed stable pelvic and lung masses and no evidence of recurrent disease in the right ventricle. No interval growth of other masses was found. The patient remains asymptomatic 6 months after surgery.

## 2. Discussion

Uterine leiomyomas are the most common gynecological tumors in pre-menopausal woman, found in up to 30% of women older than 35 years.

Despite being histologically benign, leiomyoma has clinically malignant potential. Metastases have been reported to a number of different sites, including the spine [1–4], skull [1], rib and vertebra [5], retroperitoneum [6], parametria [7], appendix [7], lymph nodes [8, 9], and most commonly the lung [10].

Different types of extrauterine growth of benign uterine leiomyomas are described as follows: disseminated peritoneal leiomyomatosis (DPLM), retroperitoneal leiomyomatosis (RPLM), parasitic leiomyoma, BML, and intravenous leiomyomatosis (IVL) [11].

DPLM and RPLM are characterized by multiple leiomyomatous masses seen in the submesothelial tissues of the abdominopelvic peritoneum and the abdominopelvic retroperitoneum, respectively [11]. Parasitic leiomyoma is observed when occasionally, a leiomyomatous mass loses its original attachment to the uterus, becomes adherent to a surrounding structure (most commonly the broad ligament), and develops an accessory blood supply. Parasitic leiomyoma can be diagnosed on pelvic US based on their typical whorled appearance with clear visual separation from the uterus and ovaries [11]. Transvaginal US allowed the diagnosis of parasitic leiomyoma in the case of our patient and confirmed that the patient was status post hysterectomy and bilateral salpingooophorectomy. RPLM, DPLM, and parasitic leiomyoma are strictly confined to the abdomen and pelvis.

In contrast, BML and IVL can cause more distant metastases, particularly to the chest.

Intravascular leiomyomatosis is characterized by the presence of vascular invasion and extension of benign smooth muscle lesions in a worm-like manner into the pelvic and systemic veins. No macroscopic vascular invasion is found in BML. Owing to its intraluminal growth, leading to symptoms of venous obstruction [12], IVL has an aggressive clinical presentation, while BML typically has a very indolent clinical course.

In IVL, US shows vascularized thrombi within the pelvic veins and inferior vena cava. CT and MR imaging demonstrate continuity in intraluminal growth from the pelvic veins [11]. None of these features were demonstrated in our patient, supporting the diagnosis of BML as opposed to IVL.

Pathogenesis of BML is controversial [13]. However, although no vascular invasion can be identified clinically or on imaging in BML, several works support microscopic vascular invasion as the metastatic mechanism of BML [14, 15] suggesting a unified pathogenesis to both IVL and BML by hematogenous spread.

While several cases of cardiac metastases have been reported with IVL [12, 16], to our knowledge, this is only the 5th case of BML metastasizing to the heart reported in the literature [17–20]. All previously reported cases had a history of hysterectomy up to 16 years prior to cardiac metastases. While 2 patients had dyspnea at the time of presentation [17, 19], 3 other patients including ours denied any cardiac symptoms and were only positive for a systolic cardiac murmur [18, 20]. Our case is the first case of BML to the heart found fortuitously on imaging and the youngest patient at the time of diagnosis of the cardiac mass.

The mass initially identified on the chest CT scan was erroneously thought to be a thrombus, leading to initiation of anticoagulation. Thrombus accounts for the most commonly encountered intracardiac mass and typically appears on CT scan as a hypodense, low attenuation filling defect, similar to intracardiac tumors [21]. While CT is not an adequate exam for intracardiac tumors, TEE is the initial diagnostic imaging workup for a cardiac mass but is operator dependent and can sometimes be diagnostically limited by its reliance on the anatomic appearance of the mass. Cardiac magnetic resonance (CMR) has become the gold standard for evaluation of such masses and allows optimal tissue differentiation and accurate characterization of the mass preoperatively [17, 20]. CMR enables the differentiation of intracardiac thrombus from a tumor due to avascular tissue composition. Early gadolinium enhancement (EGE) imaging is the ideal technique. Thrombus manifests an absence of gadolinium uptake and appears almost black on EGE imaging [22]. T1- and T2-weighted signals vary depending on the age of the thrombus. BML typically displays intermediate signal intensity on T1-weighted images, low signal intensity on T2-weighted images, and homogenous contrast enhancement [11, 22]. Interestingly, PET-CT scan also provides helpful diagnostic information as BML, like other benign tumors, has low metabolic activity with faint or nonavid FDG uptake characterized by SUVmax lower than 2.5, in contrast to malignant lesions [23, 24].

Uterine leiomyomatosis is highly hormone sensitive, and treatments are based on hormonal manipulation [25–27] with either surgical or medical castration [28, 29]. Hormone suppression has been shown to either stabilize or even induce regression of metastatic lesions [30].

However, medical treatment alone is highly insufficient in the case of intracardiac tumors. This is related to the risk of heart failure and possible sudden death caused by total outflow tract obstruction. Regardless of the pathogenesis of intracardiac leiomyomatosis, review of the literature suggests that surgical removal of the intracardiac tumor is curative [16, 31]. Complete removal is strongly recommended, as no recurrence has been reported with total resection, as opposed to 1/3 recurrence rate in patients who underwent partial resection [16]. This is regardless of postoperative antiestrogen therapy.

This case underlines the importance of considering BML as a potential differential diagnosis in any female patient with a history of hysterectomy who presents with an intracardiac tumor.

## Supplementary Materials

Supplementary Materials Transesophageal echocardiogram showing a large mobile mass in the right ventricle arising from the interventricular septum with turbulent flow in the right ventricular outflow tract.
